# Hummingbird migration and flowering synchrony in the temperate forests of northwestern Mexico

**DOI:** 10.7717/peerj.5131

**Published:** 2018-07-06

**Authors:** Gabriel López-Segoviano, Maribel Arenas-Navarro, Ernesto Vega, Maria del Coro Arizmendi

**Affiliations:** 1 Posgrado en Ciencias Biológicas, Unidad de Posgrado, Coordinación del Posgrado en Ciencias Biológicas, Universidad Nacional Autónoma de México, Coyoacán, Ciudad de México, Mexico; 2 Instituto de Investigaciones en Ecosistemas y Sustentabilidad, Universidad Nacional Autónoma de México, Morelia, Michoacán, Mexico; 3 Laboratorio de Ecología, Unidad de Biotecnología y Prototipos, Universidad Nacional Autónoma de México, Tlalnepantla, Estado de México, Mexico

**Keywords:** Hummingbird migration, Flowering phenology, *Selasphorus rufus*, *Amazilia beryllina*, Local migration, Altitudinal migration

## Abstract

**Background:**

Many species of birds are morphologically and physiologically adapted for migration. Migratory movements of birds can range from thousands of kilometers, such as when birds migrate from wintering to breeding sites in summer, to several kilometers, such as when birds migrate among habitats in a single mountain system. The main factor that influences bird migration is the seasonal fluctuation of food resources; climate, predation, competition for resources and endogenous programming are also important factors. Hummingbirds are highly dependent on nectar, so their migration is likely correlated with the blooming of plant species. The ecological implications of altitudinal migration in the mountains of North America as well as the latitudinal migration of *Selasphorus rufus* through Mexico are still poorly understood. To explore these issues, over three non-consecutive years, we evaluated interannual variation in the phenologies of a latitudinal migrant (*S. rufus*) and an altitudinal migrant (*Amazilia beryllina*) and their visited plants.

**Methods:**

We assessed the relationship between two migratory hummingbirds and flower abundance in 20 fixed-radius plots (25 m radius). All available flowers were counted along transects (40 × 5 m) inside each fixed-radius plot. Sampling was performed every 10 days from November 12 through February 20 of 2010–2011, 2013–2014 and 2015–2016, resulting in a total of 11 samples of each plot per period. Phenological variation and the relationships among hummingbird abundance, flower abundance and vegetation type were evaluated using a generalized additive mixed model.

**Results:**

*S. rufus* abundance was related to sampling time in the first and third periods; this relationship was not significant in the second period. *A. beryllina* abundance was related with the sampling time over all three periods. The abundance of *S. rufus* hummingbirds was significantly related to the number of *Salvia iodantha* flowers. The abundance of *A. beryllina* hummingbirds was related to the number of *S. iodantha* and *Cestrum thyrsoideum* flowers and the total number of flowers. We found a non-significant correlation between *S. rufus* and *A. beryllina* abundance and vegetation types.

**Conclusion:**

Contrary to expectations, the long-distance migration of *S. rufus* was not consistent over the sampling periods. The migration of *S. rufus* through the study region may be altered by changes in climate, as has occurred with other species of migratory birds. In the present study, the migration of *S. rufus* was correlated with the blooming of *S. iodantha*. In comparison, the altitudinal migrant *A. beryllina* responded to the availability of floral resources but was not associated with a particular plant. The migration of this latter species in the area probably depends on multiple factors, including climatic and demographic factors, but is particularly dependent on the supply of floral resources and competition for these resources.

## Introduction

Many species of birds are morphologically and physiologically adapted for migratory movements (Newton, 2007). The main factor that influences bird migration movement is the seasonal fluctuation of food resources (Levey & Stiles, 1992; Newton, 2007; Faaborg et al., 2010); climate, predation, competition for resources and endogenous programming (related to reproduction, molting, fat deposition and migratory restlessness) are also important (Newton, 2007). A large number of bird species breed at northern latitudes in the summer and then travel thousands of kilometers to tropical wintering destinations (Newton, 2007; Faaborg et al., 2010), while others migrate locally and seasonally from high to lower altitudes (Newton, 2007). For example, nectarivorous and frugivorous species depend on the seasonality of floral and fruit resources, which are their main source of energy, and make seasonal movements at many scales following available food sources (Levey & Stiles, 1992).

Only 29 out of the 328 known hummingbird species (8.84%) are long-distance migrants (Rappole & Schuchmann, 2003). Of these, 13 inhabit North America (Rappole & Schuchmann, 2003); these species breed during the summer in Canada and the United States and then migrate southwards during autumn (Howell, 2003). Hummingbirds migrate along established migration routes and make refueling stops at flowering grounds (Phillips, 1975; Gass, 1979; Carpenter, Paton & Hixon, 1983; Carpenter et al., 1993; Calder & Contreras-Martínez, 1995; Schuchmann, 1999; Calder, 2004; Zenzal & Moore, 2016). The duration of their stay at a particular site can be as short as one day to as long as three weeks (Gass, 1979; Carpenter et al., 1993; Nemeth & Moore, 2012; Zenzal & Moore, 2016). As hummingbirds are highly dependent on floral nectar (Gass, 1979; Hixon, Carpenter & Paton, 1983; Schuchmann, 1999), their migrations are correlated with flowering phenologies (Bertin, 1982; Calder, 1987; McKinney et al., 2012).

Similar behavior can be observed among tropical hummingbirds that move up or down foothills following the blooming of their preferred plant species (Des Ganges, 1979; Arizmendi & Ornelas, 1990; Hobson et al., 2003; Tinoco et al., 2009; Fraser, Diamond & Chavarria, 2010). Rappole & Schuchmann (2003) define altitudinal migration as the seasonal movement of a species with a home range that shifts over a distance of <10 km; altitudinal migrants generally return on a seasonal basis to their site of origin. These authors suggested that 87 hummingbird species make altitudinal migrations (26.52% of known species). These migratory movements of hummingbird species occur throughout different mountain systems of America (Des Ganges, 1979; Stiles, 1985; Des Ganges, 1979; Levey & Stiles, 1992; Hobson et al., 2003; Tinoco et al., 2009). At a local scale, altitudinal migrations are likely also related to the availability of floral resources, but birds must weigh the cost and intensity of competition for these resources (Wolf, Stiles & Hainsworth, 1976; Des Ganges, 1979).

In addition, recent climate changes can alter the timing of bird migrations (Cotton, 2003; Gordo et al., 2005; Marra et al., 2005; Saino et al., 2007; Cohen et al., 2015), resulting in an increasing mismatch between migratory birds and food resources (Both et al., 2006, 2009; Jones & Cresswell, 2010). Similarly, food resources can be influenced by climatic events, thus affecting the availability of resources for migratory birds (Visser, Holleman & Gienapp, 2006; Reed, Jenouvrier & Visser, 2013). Such phenomena can negatively affect migratory bird populations (Both et al., 2006, 2009; Jones & Cresswell, 2010).

We studied two hummingbird species: one latitudinal migrant, *Selasphorus rufus* (Healy & Calder, 2006), and one altitudinal migrant, *Amazilia beryllina* (Des Ganges, 1979; Arizmendi, 2001). *S. rufus* breeds in the Pacific Northwest of the United States and Canada and, during winter, migrates from the southwestern United States through central Mexico (Healy & Calder, 2006). Like other species of hummingbirds (Kodric-Brown & Brown, 1978; McKinney et al., 2012; Nemeth & Moore, 2012; Graham et al., 2016; Zenzal & Moore, 2016) and songbirds (Moore et al., 2017), *S. rufus* requires refueling stops in different places along its flyway (Gass, 1979; Carpenter, Paton & Hixon, 1983; Carpenter et al., 1993; Calder, 2004). The arrival of *S. rufus* at stopover sites is correlated with the blooming of its feeding plants (Calder, 1987; Kodric-Brown & Brown, 1978; Russell et al., 1994).

*A. beryllina* is most commonly found between 500 and 1,800 masl (Weller & Kirwan, 2017). In the study region, *A. beryllina* is common and abundant at mid-mountain ranges at around 1,000 masl (López-Segoviano, 2018). Des Ganges (1979) stated that *A. beryllina* is an opportunistic species that follows the blooming of feeding plants; this species may also be more sensitive than resident species to variations in the availability of nectar. *A. beryllina* are larger and heavier than *S. rufus* and migrate to the upper ranges of mountains in the study region during fall/winter (López-Segoviano, Bribiesca & Arizmendi, 2018). In addition, *A. beryllina* exhibits an intermediate level of aggressive dominance, while *S. rufus* has a low level of dominance at the study site (López-Segoviano, Bribiesca & Arizmendi, 2018). So, *S. rufus* is subordinate to *A. beryllina* (López-Segoviano, Bribiesca & Arizmendi, 2018).

The ecological implications of altitudinal migrations in the mountains of North America (Boyle, 2017) as well as the latitudinal migration of *S. rufus* through Mexico (Schondube et al., 2004) are still poorly understood. Therefore, we evaluated interannual variation in the phenologies of the *S. rufus* and *A. beryllina* hummingbird species and their visited plants in three nonconsecutive years. For *S. rufus*, we expected to find a consistent pattern in its migratory phenology because long-distance migrants are more influenced by endogenous rhythms in comparison to short-distance migrants (Newton, 2007). For *A. beryllina*, we expected to find a more variable pattern in its migratory phenology, as this species is likely influenced by local flowering and by the abundances of other hummingbird species in the local assemblage.

## Methods

### Study area

**Figure 1:**
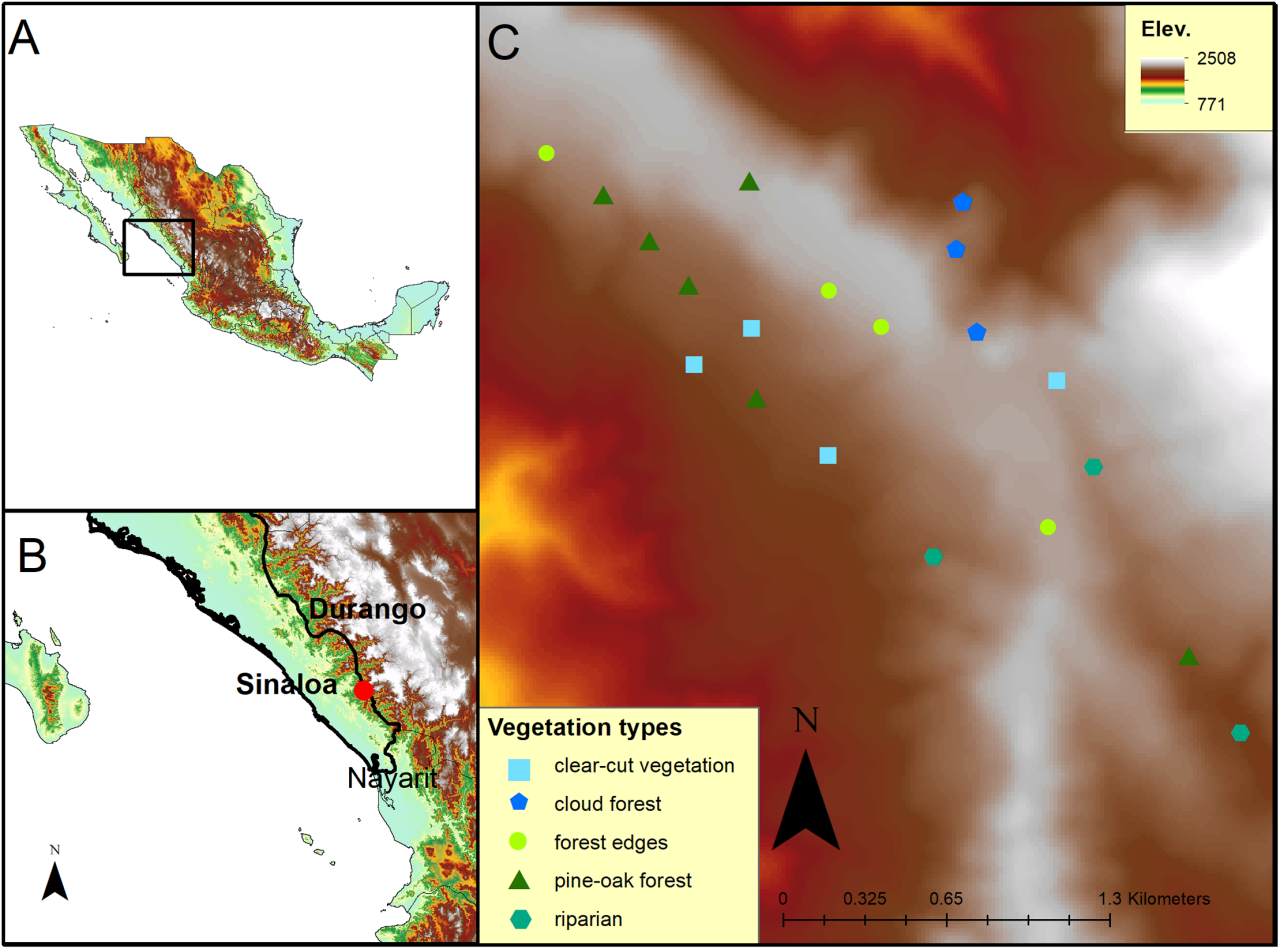
Map of the study site at El Palmito in Sinaloa, Mexico. (A) Location of the study site in Northwestern Mexico. (B) Location of the study site at El Palmito, Sinaloa. (C) Location of plots at study site; different symbols represent distinct vegetation types.

The study site was located along a western slope of the Sierra Madre Occidental mountain range at the El Palmito Concordia ejidal lands (23°34′16″ N; 105°50′15″ W) in Sinaloa, Mexico, between 1,800 and 2,200 masl (Fig. 1B). The climate is temperate sub-humid with an average annual precipitation of 1,247 mm (SMN, 2018). The Sierra Madre Occidental is the longest and most continuous mountain range in Mexico and represents an important temperate forest corridor (González-Elizondo et al., 2012). A vegetation gradient of oak forest, pine-oak forest and cloud forest mixed with riparian areas and secondary forest is present at the study site (Díaz, 2005).

A total of 14 hummingbird species have been described for the region: five residents (*Hylocharis leucotis*, *Lampornis clemenciae*, *Eugenes fulgens*, *Selasphorus platycercus* and *Atthis heloisa*), four altitudinal migrants (*Amazilia violiceps*, *A. beryllina*, *Cynanthus latirostris* and *Colibri thalassinus*) and five latitudinal migrants (*Selasphorus rufus*, *S. sasin*, *S. calliope*, *Calypte costae* and *Archilochus colubris*; López-Segoviano, 2012).

### Hummingbird censuses

To determine the migratory phenology of the studied hummingbirds, we counted individuals in 20 fixed-radius plots (25 m radius) separated by at least 188 m (minimum distance between two plots; mean distance = 332.42 m; SD = 116.33 m). At the center of each plot, all detected hummingbirds were counted for 10 min. The plots were located in a 300 ha area covered with different types of vegetation (six plots with pine-oak forest, three plots with cloud forest, four plots with forest edges, four plots with clear-cut secondary vegetation and three plots with riparian vegetation; Fig. 1C). The plots were fixed and distributed to represent the heterogeneity of the study site (Fig. 1C). All plots were sampled every 10 days from November 12 to February 20 in 2010–2011, 2013–2014 and 2015–2016, resulting in a total of 11 samples of each plot per sampling period.

We followed all recommended ethical guidelines to avoid harming hummingbird species and other animals in the research area and to minimize any effects on the environment (Fair, Ellen & Jones, 2010).

We obtained permits from the Sub-Secretariat for Environmental Protection Management, General Directorate for Wildlife (Subsecretaría de Gestión para la Protección Ambiental, Dirección General de Vida Silvestre; permit numbers SGPA/DGVS/01833/11 and SGPA/DGGFS/712/1289/16) of Mexico. The collection permit allowed voucher specimens of plants to be collected for identification by specialists.

### Flower censuses

To evaluate flower availability, all flowers inside the fixed-radius plots used for bird counts were counted along transects of 40 m in length and 5 m in width. These transects intersected the center of each plot and were oriented toward the direction where the majority of flowers within the plot were found. The abundance and identity of all flowers were recorded. Floral censuses were carried out at the same frequency as the bird counts: 11 times per period for each plot.

### Statistical analysis

The migratory phenologies and the relationship between hummingbird and flower abundances were analyzed using a generalized additive mixed model (GAMM). We used the numbers of *A. beryllina* and *S. rufus* species in each plot as the response variable and time, vegetation type (Fig. 1C) and the numbers of *S. iodantha* and *C. thyrsoideum* flowers and total flowers (total flowers of 15 plant species) as the predictor variables. Time was measured from 0 to 100 days where day 0 was the initial sampling date on November 12 and day 100 was the final sampling date on February 20. We fitted a GAMM with a Poisson (*S. rufus*) and Quasipoisson (*A. beryllina*) distribution, and the plots were incorporated as a random effect (Crawley, 2007; Zuur et al., 2009). We analyzed the model using the package mgcv (Wood, 2009) in R software version 3.3.3 (R Core Team, 2017).

## Results

### Migratory phenology

**Figure 2:**
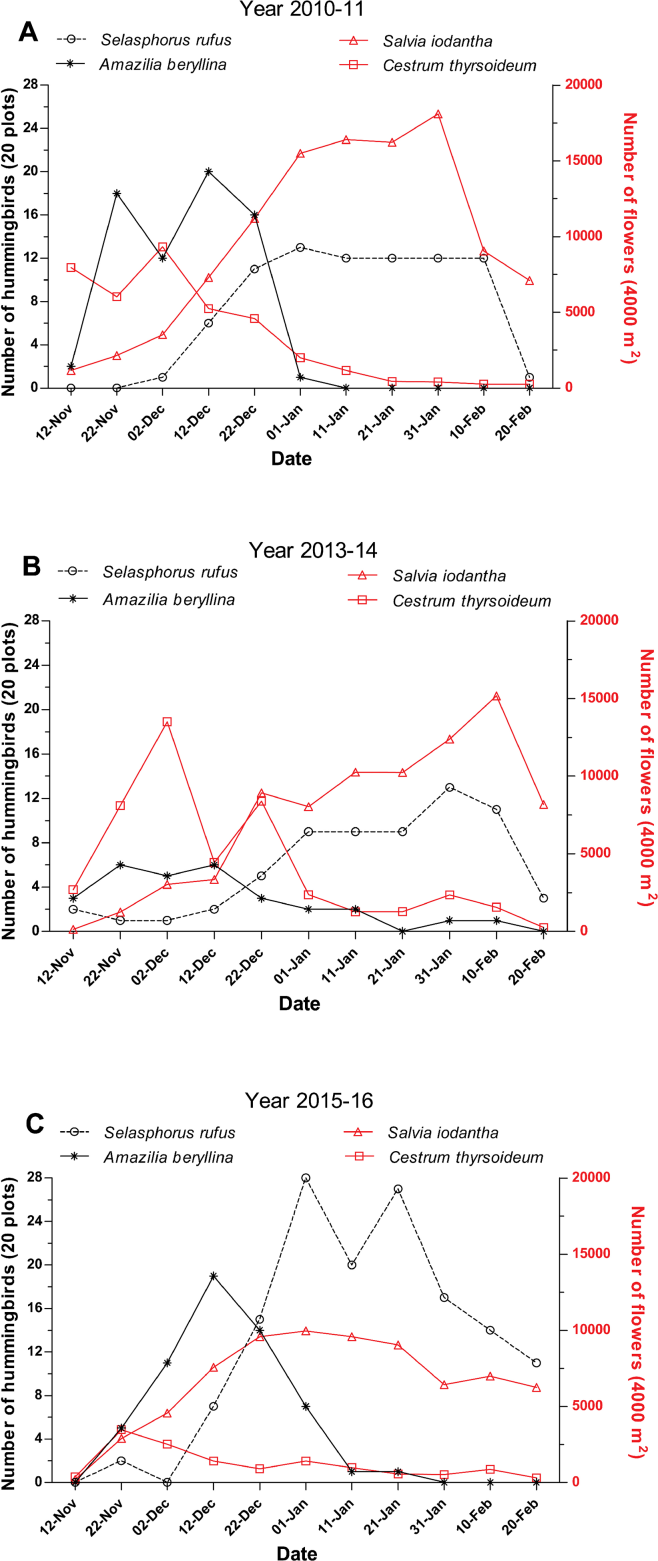
Abundance of hummingbirds *S. rufus* and *A. beryllina* and of flowers *S. iodantha* and *C. thyrsoideum* during the following sampling periods. (A) 2010–11, (B) 2013–14 and (C) 2015–16. The total of numbers of *S. rufus* (open black circle), *A. beryllina* (black asterisk), flowers of *S. iodantha* (open red triangle) and flowers of *C. thyrsoideum* (open red square) are shown.

In the three studied periods, *A. beryllina* and *S. rufus* were abundant in the region and were only surpassed in abundance by the resident species *H. leucotis. Selasphorus rufus* was the second most abundant species in the region, representing 10.6% of the total hummingbirds recorded during the first sampling period, 12.2% during the second sampling period and 20.4% during the third sampling period (Table S1). *A. beryllina* was the third most abundant species in the region during the first and third sampling periods (representing 9.1% and 8.4%, respectively, of the total hummingbirds observed) and the fourth most abundant species (representing 5.4% of the total hummingbirds observed) during the second sampling period (Table S1). The peak of abundance of *A. beryllina* and *S. rufus* were separate during the three studied periods; *A. beryllina’s* peaks of abundance occurred first followed by the peak of abundance of *S. rufus* (Fig. 2). The GAMM showed that the abundance of *S. rufus* was related with the time of sampling during the first and third periods (Table 1; Fig. 2), yet this relationship was not significant for the second period (Table 1). During 2013–2014 *S. rufus* arrived earlier and maintained low numbers and high variation during all the winter (Fig. 2). Notably, a comparatively higher level of precipitation was recorded during the second period (Table S2). The abundance of *A. beryllina* was related with the time of sampling over all three periods (Table 1).

**Table 1:** Results from the generalized additive mixed model (GAMM) to assess relationships between the abundances of *S. rufus* and *A. beryllina* and flowering plants (*S. iodantha*, *C. thyrsoideum* and total number of flowers), time per sampling.

	*S. rufus*			*A. beryllina*		
	df/edf	*F*	*P*	df/edf	*F*	*P*
Vegetation	4	0.46	0.765	4	0.947	0.436
s(Times):Period 1	2.375	3.408	0.037	6.783	46.25	<0.001
s(Times):Period 2	1.000	0.111	0.738	1.000	95.85	<0.001
s(Times):Period 3	3.775	19.656	<0.001	4.119	59.37	<0.001
s(*S. iodantha*)	6.405	9.932	<0.001	1.648	11.87	<0.001
s(*C. thyrsoideum*)	1.000	0.884	0.347	1.000	20.50	<0.001
s(Total flowers)	1	1.207	0.272	6.436	23.02	<0.001

**Note:**

Later, the ANOVA command was used to clarify the significance of the individual terms (Crawley, 2007). df, degrees of freedom; edf, effective degrees of freedom for the spline function.

### Flowering synchrony

**Figure 3:**
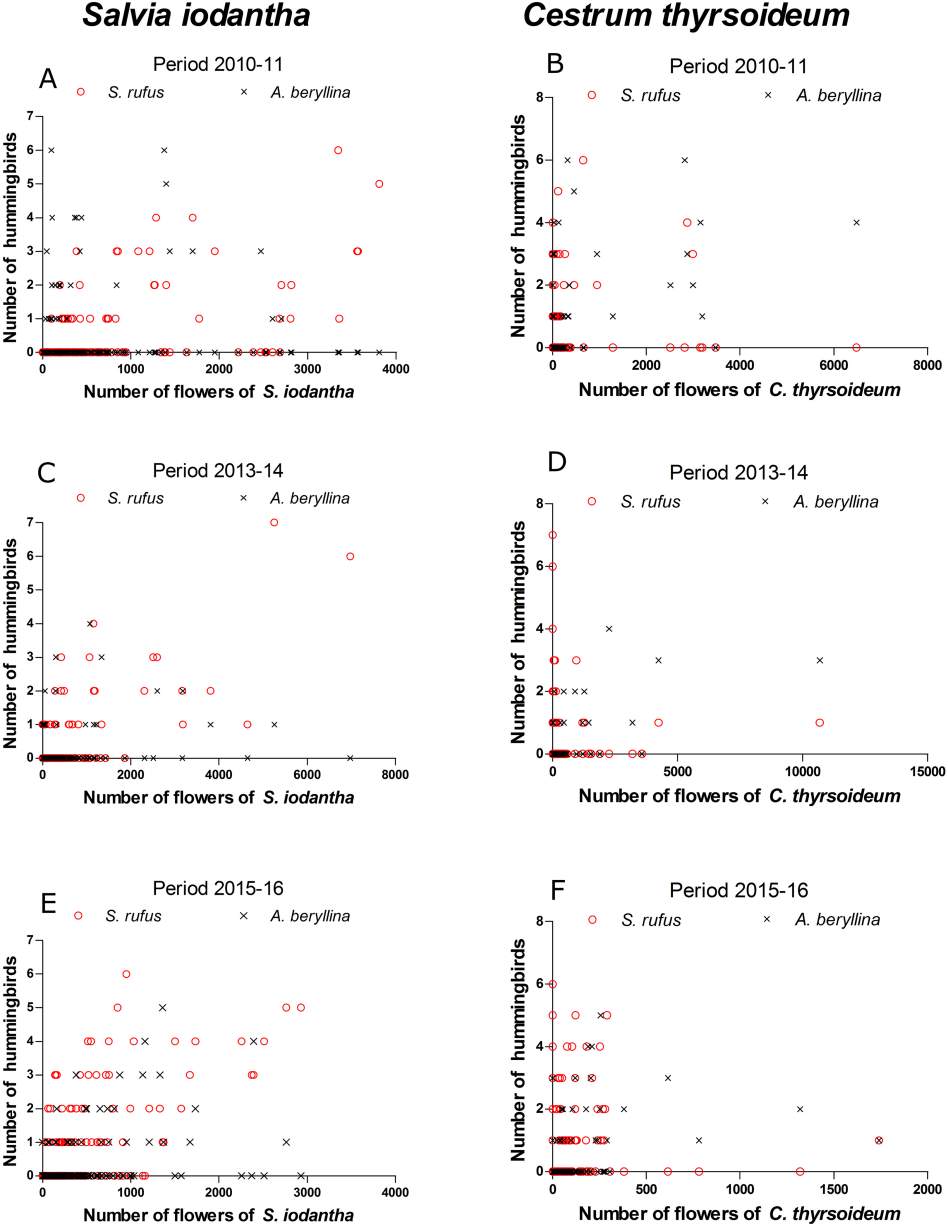
Scatter plots of the number of *S. rufus* (open red circle) and *A. beryllina* (black cross) and the number of flowers of *S. iodantha* and *C. thyrsoideum* during the following sampling periods. *S. iodantha*: (A) 2010–11, (C) 2013–14 and (E) 2015–16; *C. thyrsoideum*: (B) 2010–11, (D) 2013–14 and (F) 2015–16.

We registered 15 plant species at the phenological transects. *S. iodantha* and *C. thyrsoideum* were the most abundant species. Over the three sampling periods, *S. iodantha* represented 69%, 61% and 77%, respectively, of total flowers counted in the region, followed by *C. thyrsoideum*, which represented 24%, 35% and 14%, respectively, of total flowers (Table S3). The flowering phenology of *S. iodantha* was similar during each sampling period and corresponded with the arrival of *S. rufus* to the study site; *S. rufus* tended to follow the flowering of *S. iodantha*, a pattern that repeats each sampling period (Fig. 2). Meanwhile, *A. beryllina* arrival to the study site much earlier than *S. rufus* (Fig. 2); in the first period *A. beryllina* peak of abundance were when *C. thyrsoideum* flowering occurred, in the second period it followed *C. thyrsoideum* weakly. *C. thyrsoideum* presented a distinct blooming tendency in third sampling period. The flowering of *C. thyrsoideum* was almost finished when *S. rufus* presented its peak of abundance in each sampling period (Fig. 3).

According to the GAMM models, a significant correlation was found between the number of *S. rufus* hummingbirds and the number of *S. iodantha* flowers (Table 1; Fig. 3). A non-significant correlation was found between the number of *S. rufus* hummingbirds and the number of *C. thyrsoideum* flowers and total flowers (Table 1; Fig. 3). Also, a non-significant correlation was found between the number of *S. rufus* hummingbirds and the vegetations type in each plot (Table 1). *A. beryllina* was related to the number of *S. iodantha* flowers, *C. thyrsoideum* (Fig. 3) flowers and total number of flowers (Table 1). Finally, we found a non-significant correlation between the number *A. beryllina* hummingbirds and the vegetation types in each plot (Table 1).

## Discussion

### Migratory phenology

Our study showed variation in the relationship between the abundance of *S. rufus* and the sampling date during the second period, while a constant relationship was found for *A. beryllina* over all three sampled periods. This result contrasts with those of Supp et al. (2015) where long-migration hummingbird species like *S. rufus* were found to have migratory periods with lower interannual variation in comparison to hummingbird species with shorter migratory routes. However, variation in climatic conditions can affect the migration times of some bird species (Cotton, 2003; Gordo et al., 2005; Marra et al., 2005; Saino et al., 2007). Marra et al. (2005) suggested that variation in spring temperatures influences the migration of long-distance migratory birds; in this study, birds were found to migrate earlier in warm years and later in colder years. The environmental conditions (i.e., precipitation) varied in the second period of our study, yet the change in the migration pattern of *S. rufus* may also be the result of variation in local environmental conditions at its breeding sites.

Meanwhile, altitudinal migrant hummingbirds may perform movements of only a few kilometers but can search for resources along an altitudinal gradient. Generally, altitudinal migration is optional in the short term; sedentary species might migrate, for example, to avoid periods of adverse weather (reviewed by Faaborg et al., 2010). The altitudinal migrant *A. beryllina* examined in our study may migrate depending on local climate conditions and resource quality. Several additional studies establish that seasonal variation of food resources is the main factor that influences the altitudinal migration of birds (Levey & Stiles, 1992; Newton, 2007; Faaborg et al., 2010; Boyle, 2017). For altitudinal migrant hummingbirds, the availability of food resources as well as competition with other hummingbirds for shared resources is an important factor (Wolf, Stiles & Hainsworth, 1976; Des Ganges, 1979). Wolf, Stiles & Hainsworth (1976) stated that dominance interactions and floral availability influence the migration of altitudinal migrant hummingbirds. Meanwhile, Rappole & Schuchmann (2003) proposed that hummingbird migrations respond to seasonal scarcity of resources as well as seasonal flushes of resources at other sites. However, more studies are needed to determine the importance of competition for resources and climatic conditions for *A. beryllina*’s altitudinal migration.

Furthermore, annual variation in the climatic conditions of winter sites could decouple birds from their usual migratory phenology (Cotton, 2003; Saino et al., 2007). If migratory hummingbirds are unable to adjust their migration to specific flowering dates or shortened flowering duration of their preferred plants along their migratory routes, these hummingbirds will be less successful, and their populations will likely be reduced (Faaborg et al., 2010). Thus, the decoupling of migrants and food resource availability along migratory routes can have direct consequences for the state of migratory populations (Both et al., 2006, 2009; Jones & Cresswell, 2010). For example, Reed, Jenouvrier & Visser (2013) found such a mismatch can have strong effects on the relative fitness and egg-laying dates of the migratory bird *Parus major* (Great Tits) for several years, although a weak effect was found for mean demographic rates. However, population decline as a result of phenological mismatching cannot be considered as a common process affecting all migratory bird species, as this may depend on multiple factors such as migration distance, continent and habitat seasonality (Both et al., 2009; Jones & Cresswell, 2010).

### Flowering synchrony

Our study found a relationship between the number of *S. rufus* migratory birds and the number of *S. iodantha* flowers. As the migration of *S. rufus* is the longest of all migrating hummingbirds in North America (Supp et al., 2015), the coupling of its migratory route with a diverse assemblage of blooming plant species is expected (Calder, 1987; Kodric-Brown & Brown, 1978; Russell et al., 1994). In this study, the presence of *S. rufus* was coupled with the flowering of *S. iodantha* in northwestern Mexico; this was also found in another area of western Mexico (Manantlán, Jalisco) where *S. rufus* was the most abundant migratory hummingbird in winter and visited *S. iodantha* flowers (vs. other flowers) more frequently (Arizmendi, 2001). This confirms the importance of the flowering phenology of *S. iodantha* for the fall migration of *S. rufus* along its migratory route in western Mexico. This can be considered equivalent to the role of *Impatiens biflora* flowers for the fall migration of the Ruby-Throated Hummingbird (*Archilochus colubris*); the peak in flowering times of *I. biflora* is closely related to the peak migration time of the Ruby-Throated Hummingbird throughout the eastern United States (Bertin, 1982). However, recent studies found that the correlation in phenology between Ruby-Throated Hummingbirds and *I. biflora* is not supported in southern breeding individuals in United States (Zenzal et al., 2018).

In this respect, migratory species’ selection of refueling sites directly influences their survival. In an unknown environment, migratory species have limited time and energy to sample the habitat and experience greater susceptibility to predation and increased competition (McGrath, van Riper & Fontaine, 2009). In response, *S. rufus* has been shown to establish territories that exclude other hummingbird species along its migratory route in the United States to gain priority access to food resources (Gass, 1979; Kodric-Brown & Brown, 1978; Kuban & Neill, 1980). However, in Mexico, local hummingbird species have larger body sizes (including *A. beryllina*) and dominate smaller latitudinal migratory species, displacing them to floral patches with less rewarding resources (Des Ganges, 1979; Calder & Contreras-Martínez, 1995; Rodríguez-Flores & Arizmendi, 2016; López-Segoviano, Bribiesca & Arizmendi, 2018). For this reason, *S. rufus* individuals prefer to feed on floral patches of *S. iodantha*; these flowers do not provide maximum energy quality but are available to *S. rufus* because more dominant hummingbird species prefer other resources (López-Segoviano, Bribiesca & Arizmendi, 2018). This synchrony between the latitudinal migration of *S. rufus* and flowering phenology may also be present at other sites along the migration route of *S. rufus* in Mexico (Calder & Contreras-Martínez, 1995).

Regarding abundances, we found that *A. beryllina* abundance was related to the availability of floral resources in general (*S. iodantha*, *C. thyrsoideum* and total number of flowers) in the study area. This confirms that altitudinal migratory hummingbirds primarily respond to variability in the supply of local floral resources (Stiles, 1985). During periods with less abundant floral resources, hummingbird species respond by performing altitudinal or partial migrations to areas with better supplies of floral resources (Stiles, 1985). Thus, hummingbird communities change depending on the availability of local floral resources (Feinsinger, 1976; Arizmendi & Ornelas, 1990; Cotton, 2007). This is especially evident in species with short altitudinal migrations, such as *A. beryllina*, which can navigate through regions with different vegetation types and climate. However, it is necessary to perform further studies on the additional factors that influence the migration of *A. beryllina* such as biotic interactions (e.g., competition among species) and abiotic factors (e.g., climatic conditions).

Finally, we did not find a relationship between the number of *S. rufus* and *A. beryllina* hummingbirds and different vegetation types. Many temperate forests in Mexico have been clear-cut; some of these areas are now regenerating, resulting in secondary vegetation with abundant plants for hummingbirds to feed on. In some cases, secondary vegetation may even have more available flowers than pristine vegetation (Calder & Contreras-Martínez, 1995; Rodríguez-Flores & Arizmendi, 2016). Rodríguez-Flores & Arizmendi (2016), for example, found more *A. beryllina* and *S. rufus* individuals in secondary vegetation than in pine forest. Likewise, we found *S. iodantha* and *C. thyrsoideum* flowers in all vegetation types but to a greater extent in clearings with secondary vegetation. Even so, we did not find that vegetation type was important for the abundance of the studied hummingbird species. In another study, Cohen, Moore & Fischer (2012) translocated and released migrant songbirds in different forested habitat types during their spring migration; these authors found that migrants explore the habitat the morning after release and move further in habitat types characterized by reduced food resources. They also suggested that migrant songbirds may search for areas with sufficient food as opposed to areas with the most abundant food supply (Cohen, Moore & Fischer, 2012).

## Conclusion

Contrary to expectations, the migration of the long-distance migratory hummingbird *S. rufus* was not consistent over the sampled periods. During migratory movements, birds decide where to stop over in response to a combination of endogenous and exogenous factors (Cohen, Moore & Fischer, 2012). The migration of *S. rufus* through the study region can be altered by changes in climate, as has been demonstrated for other species of migratory birds (Cotton, 2003; Gordo et al., 2005; Marra et al., 2005; Saino et al., 2007); however, long-term data are necessary to establish that changes in migratory patterns are associated with changes in climate. In our study, the presence of *S. rufus* coincided with the blooming of *S. iodantha*, although this was not the case for the altitudinal migratory species *A. beryllina*. Furthermore, *S. rufus* feeds more on *S. iodantha* flowers than on *C. thyrsoideum* flowers (López-Segoviano, Bribiesca & Arizmendi, 2018). In contrast, *A. beryllina* was not associated with a particular plant, as suggested by Des Ganges (1979) at another study site, but responded to the overall availability of floral resources. The migration of this latter altitudinal migratory species in the area likely depends on the supply of floral resources and competition for such resources in addition to multiple other factors, including climatic and demographic factors. More studies are needed to clarify the migratory patterns of *A. beryllina* throughout the mountains of Mexico.

## Supplemental Information

10.7717/peerj.5131/supp-1 Supplemental Information 1 Table S1. Hummingbird species, migratory status, number of observed hummingbird and percentage of observed hummingbird for the three years sampled.

10.7717/peerj.5131/supp-2 Supplemental Information 2 Table S2. Climate data and meteorological station names. Max Temperature, Min Temperature, Mean Temperature and coefficient of variation per sampling period corresponding period (November–February). Max Precipitation, Min Precipitation, Mean Precipitation and coefficient of variation per sampling period corresponding period (November–February). Meteorological stations names and coordinates. The data of each meteorological station are available at http://smn.cna.gob.mx.

10.7717/peerj.5131/supp-3 Supplemental Information 3 Table S3. Plant species, family, number of flowers and percentage of flowers for the three years sampled.

10.7717/peerj.5131/supp-4 Supplemental Information 4 Raw dataset.

## Additional Information and Declarations

### Data Availability

The following information was supplied regarding data availability: The raw data are provided as a Supplemental File.
