# Supplementary material for: Hummingbird migration and flowering synchrony in the temperate forests of northwestern Mexico
Source: PeerJ. 2018 Jul 6;6:e5131. doi: 10.7717/peerj.5131 (PMC6037137; doi:10.7717/peerj.5131)
Supplement: Supplemental Information 1 [file peerj-06-5131-s001.docx]

**Table S1**. Hummingbird species, migratory status, number of observed hummingbird and percentage of observed hummingbird for the three periods sampled (November-February).

| Scientific name | Migratory status | Year 2010-11 | | Year 2013-14 | | Year 2015-16 | | |
| --- | --- | --- | --- | --- | --- | --- | --- | --- |
|  |  | n | % | n | % | n | % | |
| *H. leucotis* | Resident | 520 | 68.78 | 356 | 66.66 | 379 | | 54.93 |
| *S. rufus* | Migratory | 80 | 10.58 | 65 | 12.17 | 141 | | 20.43 |
| *A. beryllina* | Migratory altitudinal | 69 | 9.13 | 29 | 5.43 | 58 | | 8.41 |
| *L. clemenciae* | Resident | 33 | 4.37 | 50 | 9.36 | 53 | | 7.68 |
| *C. costae* | Migratory | 17 | 2.25 | 1 | 0.19 | 1 | | 0.14 |
| *C. thalassinus* | Migratory altitudinal | 11 | 1.46 | 6 | 1.12 | 27 | | 3.91 |
| *C. latirostris* | Migratory altitudinal | 9 | 1.19 |  |  | 2 | | 0.29 |
| *A. violiceps* | Migratory altitudinal | 7 | 0.93 | 1 | 0.19 | 1 | | 0.14 |
| *S. calliope* | Migratory | 4 | 0.53 | 2 | 1.87 |  | |  |
| *S. platycercus* | Resident | 3 | 0.40 | 10 | 1.57 | 16 | | 2.32 |
| *A. colubris* | Migratory | 2 | 0.26 | 3 | 0.56 | 2 | | 0.29 |
| *E. fulgens* | Resident | 1 | 0.13 | 6 | 1.12 | 3 | | 0.43 |
| *A. heloisa* | Resident |  |  | 5 | 0.94 | 7 | | 1.01 |
| **Total** |  | **756** |  | **534** |  | **690** | |  |
