# Supplementary material for: Hummingbird migration and flowering synchrony in the temperate forests of northwestern Mexico
Source: PeerJ. 2018 Jul 6;6:e5131. doi: 10.7717/peerj.5131 (PMC6037137; doi:10.7717/peerj.5131)
Supplement: Supplemental Information 2 — Max Temperature, Min Temperature, Mean Temperature and coefficient of variation per sampling period corresponding period (November–February). Max Precipitation, Min Precipitation, Mean Precipitation and coefficient of variation per sampling period corresponding period (November–February). Meteorological stations names and coordinates. The data of each meteorological station are available at http://smn.cna.gob.mx. [file peerj-06-5131-s002.docx]

**Table S2.** Climate data and meteorological stations names. Max Temperature, Min Temperature, Mean Temperature and coefficient of variation per sampling period corresponding period (November–February). Max Precipitation, Min Precipitation, Mean Precipitation and coefficient of variation per sampling period corresponding period (November–February). Meteorological stations names and coordinates. The data of each meteorological station are available at http://smn.cna.gob.mx

|  | **AT (°C)** | | | | **AP (mm)** | | | |
| --- | --- | --- | --- | --- | --- | --- | --- | --- |
|  | Max | Min | Mean | cv | Max | Min | Mean | cv |
| **2010-2011** | 22.9 | 6.4 | 15.5 | 0.3487 | 8 | 0 | 0.4 | 2.4596 |
| **2013-2014** | 26.1 | 5.4 | 15.3 | 0.4641 | 413.7 | 0 | 64.7 | 0.9961 |
| **2015-2016** | 26.6 | 3.7 | 16.4 | 0.4337 | 115.2 | 0 | 21.4 | 1.3882 |

| Station | Municipality | Code | Lat | Long | Elevation (masl) |
| --- | --- | --- | --- | --- | --- |
| Siqueiros | Mazatlán | 25119 | 23.340556 N | -106.240556 W | 40 |
| El Quemado | Mazatlán | 25176 | 23.562500 N | -106.466667 W | 50 |
| Potrerillos | Concordia | 25074 | 23.453611 N | -105.825833 W | 1572 |
| El Salto | Durango | 10025 | 23.78333 N | -105.366667 W | 2560 |
| La Peña | Pueblo Nuevo | 10038 | 23.554167 N | -105.411111 W | 2756 |
