# Supplementary material for: Hummingbird migration and flowering synchrony in the temperate forests of northwestern Mexico
Source: PeerJ. 2018 Jul 6;6:e5131. doi: 10.7717/peerj.5131 (PMC6037137; doi:10.7717/peerj.5131)
Supplement: Supplemental Information 3 [file peerj-06-5131-s003.docx]

**Table S3**. Plant species, family, number of flowers and percentage of flowers for the three periods sampled (November-February).

|  |  | | Year 2010-11 | | | | Year 2013-14 | | | | Year 2015-16 | | | |
| --- | --- | --- | --- | --- | --- | --- | --- | --- | --- | --- | --- | --- | --- | --- |
| Specie | Family | n | | % | | n | | % | | n | | % | |  |
| *Salvia iodantha* | Lamiaceae | | 107688 | | 69.080 | | 80968 | | 60.637 | | 73014 | | 77.152 | |
| *Cestrum thirsoideum* | Solanaceae | | 37636 | | 24.143 | | 46189 | | 34.591 | | 13281 | | 14.034 | |
| *Salvia elegans* | Lamiaceae | | 1528 | | 0.980 | | 1279 | | 0.958 | | 3110 | | 3.286 | |
| *Salvia mexicana* | Lamiaceae | | 1392 | | 0.893 | | 680 | | 0.509 | | 498 | | 0.526 | |
| *Cuphea hookeriana* | Lythraceae | | 969 | | 0.622 | | 396 | | 0.297 | | 396 | | 0.418 | |
| *Cuphea calcarata* | Lythraceae | | 6145 | | 3.942 | | 3508 | | 2.627 | | 1992 | | 2.105 | |
| *Psittacanthus calyculatus* | Loranthaceae | | 120 | | 0.077 | | 262 | | 0.196 | | 2024 | | 2.139 | |
| *Bouvardia ternifolia* | Rubiaceae | | 25 | | 0.016 | | 2 | | 0.001 | |  | | 0.000 | |
| *Tillandsia bourgaei* | Bromeliaceae | |  | |  | | 6 | | 0.004 | | 0 | | 0.000 | |
| *Penstemon* sp | Plantaginaceae | | 130 | | 0.083 | | 106 | | 0.079 | | 57 | | 0.060 | |
| *Salvia gesneriflora* | Lamiaceae | | 51 | | 0.033 | | 15 | | 0.011 | | 50 | | 0.053 | |
| *Lobelia laxiflora* | Campanulaceae | | 205 | | 0.132 | | 17 | | 0.013 | | 62 | | 0.066 | |
| *Fuchsia cylindracea* | Rubiaceae | |  | |  | | 40 | | 0.030 | | 14 | | 0.015 | |
| *Loeselia mexicana* | Polemoniaceae | |  | |  | | 35 | | 0.026 | |  | |  | |
| *Castilleja arvensis* | Orobanchaceae | |  | |  | | 25 | | 0.019 | | 138 | | 0.146 | |
| Total |  | | 155889 | |  | | 133528 | |  | | 94636 | |  | |
